# The neurophysiology of sensorimotor prosthetic control

**DOI:** 10.1186/s42490-024-00084-y

**Published:** 2024-10-01

**Authors:** Sherif M. Elbasiouny

**Affiliations:** 1 Department of Biomedical, Industrial and Human Factors Engineering, College of Engineering and Computer Science, Wright State University, Dayton, OH USA; 2 Department of Neuroscience, Cell Biology, and Physiology, Boonshoft School of Medicine, College of Science and Mathematics, Wright State University, Dayton, OH USA

**Keywords:** Prostheses, Closed-loop, Sensorimotor control, Bidirectional

## Abstract

Movement is a central behavior of daily living; thus lost or compromised movement due to disease, injury, or amputation causes enormous loss of productivity and quality of life. While prosthetics have evolved enormously over the years, restoring natural sensorimotor (SM) control via a prosthesis is a difficult problem which neuroengineering has yet to solve. With a focus on upper limb prosthetics, this perspective article discusses the neurophysiology of motor control under healthy conditions and after amputation, the development of upper limb prostheses from early generations to current state-of-the art sensorimotor neuroprostheses, and how postinjury changes could complicate prosthetic control. Current challenges and future development of smart sensorimotor neuroprostheses are also discussed.

## Background

Amputation entails the loss or removal of a body part, such as a hand, arm, or leg. This experience can profoundly alter one’s life, impacting mobility, independence, work capabilities, and social interactions. Additionally, challenges such as phantom limb pain and emotional distress often emerge over time after amputation and could impede recovery. While limb loss could results from many causes, the primary causes are vascular diseases, cancer, as well as trauma [1]. With 1.6 million Americans living with limb loss in 2005 (~ 185,000 new amputations annually), this number is projected to more than double (to 3.6 million) in 2050 [2].

Despite ample research on lower limb amputations, there are much fewer studies focusing on upper limb amputations in current literature [1]. While prosthetics improve aesthetics and offer hope for enhancing daily functioning for many amputees, those with upper limb loss face considerable obstacles, leading to high rejection rates of prostheses [3, 4]. Among these obstacles are poor prosthesis embodiment, decline in prosthetics’ responsiveness; thereby requiring frequent daily recalibration of their motor decoders, as well as phantom limb pain. While many research studies focus on the mechanical and control design of upper limb prostheses, much less literature discusses the neurophysiology of prosthetic control. Accordingly, this perspective article discusses the cellular neurophysiology of prosthetic control, how the cellular properties of the sensorimotor pathways change after injury, and how those could impact the prosthetic control.

## Main text

### Neural control of movement under healthy conditions

**Fig. 1:**
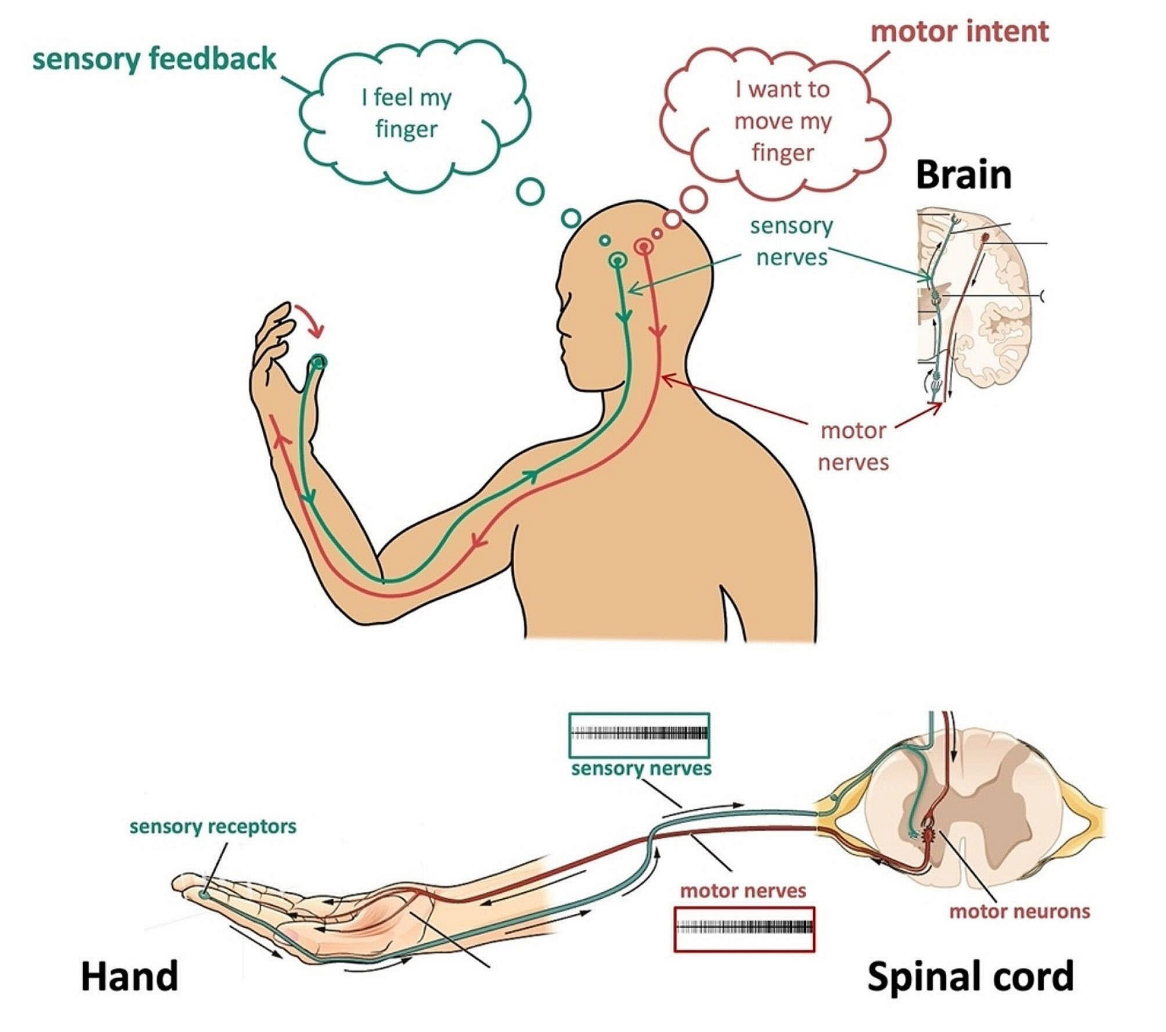
Neural control of movement under healthy conditions. Motor intent originates in the motor cortex and is transferred – via motor nerves and pathways – to activate spinal motoneurons in lamina IX of the ventral horn to eventually activate muscles. Generated movement activates sensory receptors in the periphery to send sensory information – via sensory nerves – back to spinal motoneurons and the sensory cortex, which evokes the sensation of touch

Normally, movement commands descend from the motor cortex to the sensorimotor (SM) circuit in the spinal cord (Fig. 1). SM circuits contain different types of spinal motoneurons: Small, slow S types; moderate FR types; and large, fast-fatigable FF types [5]. Spinal motoneurons are activated in increasing order of size, force production, and speed [6], allowing gradation of force and speed. Movement is also modulated by sensory afferents that return signals from muscle to motoneurons and interneurons, which process sensory and motor signals concurrently via feedback loops of mono and polysynaptic neuronal pathways. These sensory and motor feedback loops converge on the motoneuron, causing the motoneuron to receive concurrent, repeated motor and sensory synaptic signals. These sensory and motor synaptic inputs are integrated and interpreted via highly complex, nonlinear cellular processes [e.g., neurotransmitter release generates excitatory postsynaptic potentials, voltage-gated ion channels generate action potentials] to generate a modified motor command to produce graded, SM control of movement. Importantly, the brainstem, via its monoaminergic drive to the spinal cord, modulates the neuromodulatory state of the nervous system via regulating the voltage-sensitive ion channels on motoneuron and interneuron membranes via secondary messenger systems [6, 7]. This neuromodulatory state is set primarily through levels of serotonin and norepinephrine released [8, 9]. High levels of serotonin and norepinephrine leads to high neuromodulatory states, which increase the excitability of spinal motoneurons and interneurons, leading to larger synaptic inputs and high firing rates and force output, whereas low neuromodulatory states do the opposite [6, 10]. Accordingly, the brainstem dynamically regulates the excitability of the spinal SM loops, the amplitudes of their synaptic inputs on motoneurons, and the motoneuron firing rates to support multiple motor activity levels.

### Evolution of upper limb prosthetics

Advancement of prosthetics can do much to restore productivity and quality of life to amputees. Over the years, upper limb prosthetics have evolved enormously in prosthesis design, control, and functionality from just cosmetic to restoring natural SM control with the long-term goal of restoring the lost SM functionality.

### Cometic upper limb prostheses (1st generation)

**Fig. 2:**
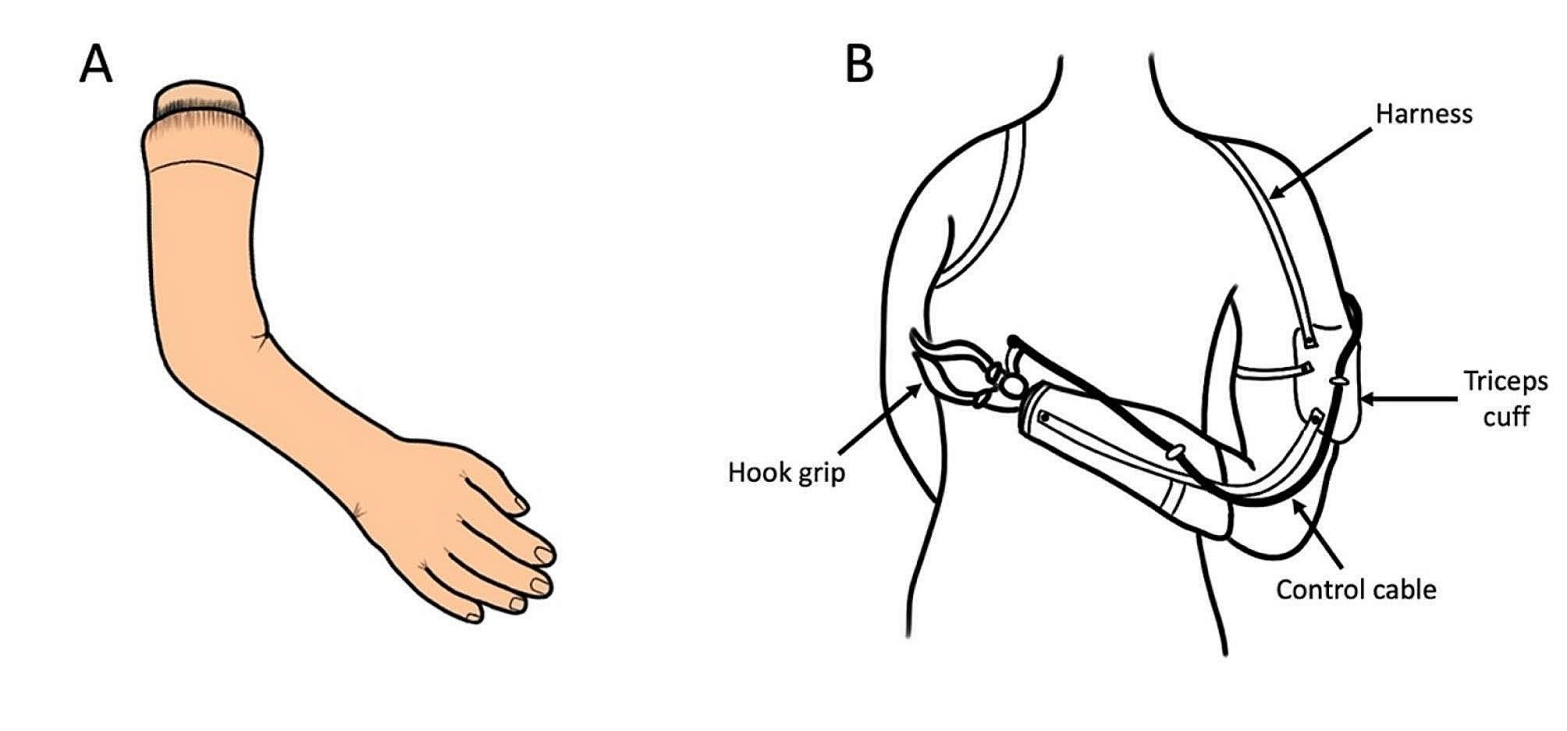
Development of upper limb prostheses. **A**) A cosmetic upper limb prosthesis. **B**) An example of a body-powered shoulder prosthesis

Cosmetic upper limb prostheses are devices designed to replicate the appearance of a missing hand, arm, or forearm with a primary focus on restoring a natural-looking aesthetic rather than providing functional capabilities like active movement or sensation (Fig. 2A). Accordingly, they are often custom-made to match the wearer’s skin tone, contours, and proportions, aiming to blend seamlessly with the remaining natural limb and to enhance the lifelike appearance [11]. They are also typically made from materials like silicone, which can be molded and colored to resemble human skin. Cosmetic prostheses can be attached using a variety of methods, such as straps, harnesses, or suction mechanisms. Although they do not provide functional abilities, cosmetic upper limb prostheses can have a positive impact on an individual’s self-esteem, body image, and overall well-being, as having a prosthesis that resembles the missing limb can help individuals feel more confident and comfortable in social and public settings [11].

### Body-powered prostheses (2nd generation)

Body-powered upper limb prostheses rely on the amputee’s body movements to operate. These prostheses typically use cables, harnesses, and mechanical linkages attached to the residual limb to translate specific movements of the amputee’s body into desired actions of the prosthesis (Fig. 2B). They are designed with different grip types (e.g., hook grips, pinch grips, and precision grips), allowing amputees to perform a range of tasks. One of the primary advantages of body-powered prostheses is their mechanical simplicity, which can lead to increased durability and reduced maintenance requirements compared to more complex electronically powered prostheses [4]. They are also reliable and cost-effective. However, body-powered upper limb prostheses are generally limited to executing basic functional movements (e.g., the opening and closing of a hand), lacking the finesse of fine motor control or sensory feedback [12]. Consequently, amputees using such prostheses often rely on specific body motions, which may not be inherently intuitive (e.g., raising or lowering the shoulder to facilitate the opening and closing of a hook grip, Fig. 2B).

### Myoelectric prostheses (3rd generation)

Myoelectric prostheses use EMG signals recorded from residual muscles on the affected side (or intact muscles on the unaffected side) to control the prosthesis. EMG electrodes record the signal when the muscle contracts, which is then processed and used to control the movements of the prosthesis. As such, amputees can control various movements of the prosthetic limb by modulating their muscle contractions. Different muscle contractions can be associated with specific actions, such as opening or closing the hand, rotating the wrist, or bending the elbow, allowing a wide range of movements. Also, features of the EMG signals could be used to provide proportional control of the prosthesis [for review, see 13]. Accordingly, myoelectric prostheses have several advantages over cosmetic and body-powered prostheses. First, EMG signals are easy to record and can provide some biological control of the prosthetic movement. Second, they could offer more advanced prosthetic control (movement speed of prosthesis is proportional to muscle contraction) and replicate a wider range of movements, allowing users to perform intricate tasks [14]. However, the myoelectric prosthetic control could be unreliable (when weak EMG signals from residual muscles on the affected side are used) or non-intuitive (when EMG signals from intact muscles on the unaffected side are used). Also, the presence of EMG crosstalk among neighboring muscles could result in unreliable prosthetic control [15]. Amputees typically need training to learn how to generate the appropriate muscle contractions to effectively control the movements of the prosthetic limb for specific actions, and myoelectric prostheses typically require frequent daily decoder calibration (to adapt to shifts in electrode placement). Despite their functional movements, myoelectric prostheses provide no sensation. Myoelectric prostheses have advanced electronics and are powered by batteries. Thus, hardware repairs and maintenance are usually more in myoelectric than body-powered prostheses.

### Targeted muscle reinnervation (TMR) prostheses (4th generation)

TMR is an invasive surgical procedure used in conjunction with myoelectric prostheses to enhance prosthetic control and provide more intuitive movement capabilities for amputees [16, 17]. In this procedure, residual motor nerves from the amputated limb are redirected to reinnervate nearby intact muscles that are still functional [18]. When the amputee attempts to move the missing limb, the reinnervated muscles generate strong and reliable EMG signals that can be used to control the prosthesis movement (via a motor decoder), allowing for more intuitive prosthetic control. Additionally, because with TMR amputees can have multiple distinct muscle signals available for control, this can allow for more complex and nuanced movement of the prosthetic limb. As with myoelectric prostheses, amputees with TMR prostheses need training and daily calibrations to learn how to effectively control the movements of the prosthetic limb [19].

### Sensorimotor (SM) neuroprostheses (5th generation)

**Fig. 3:**
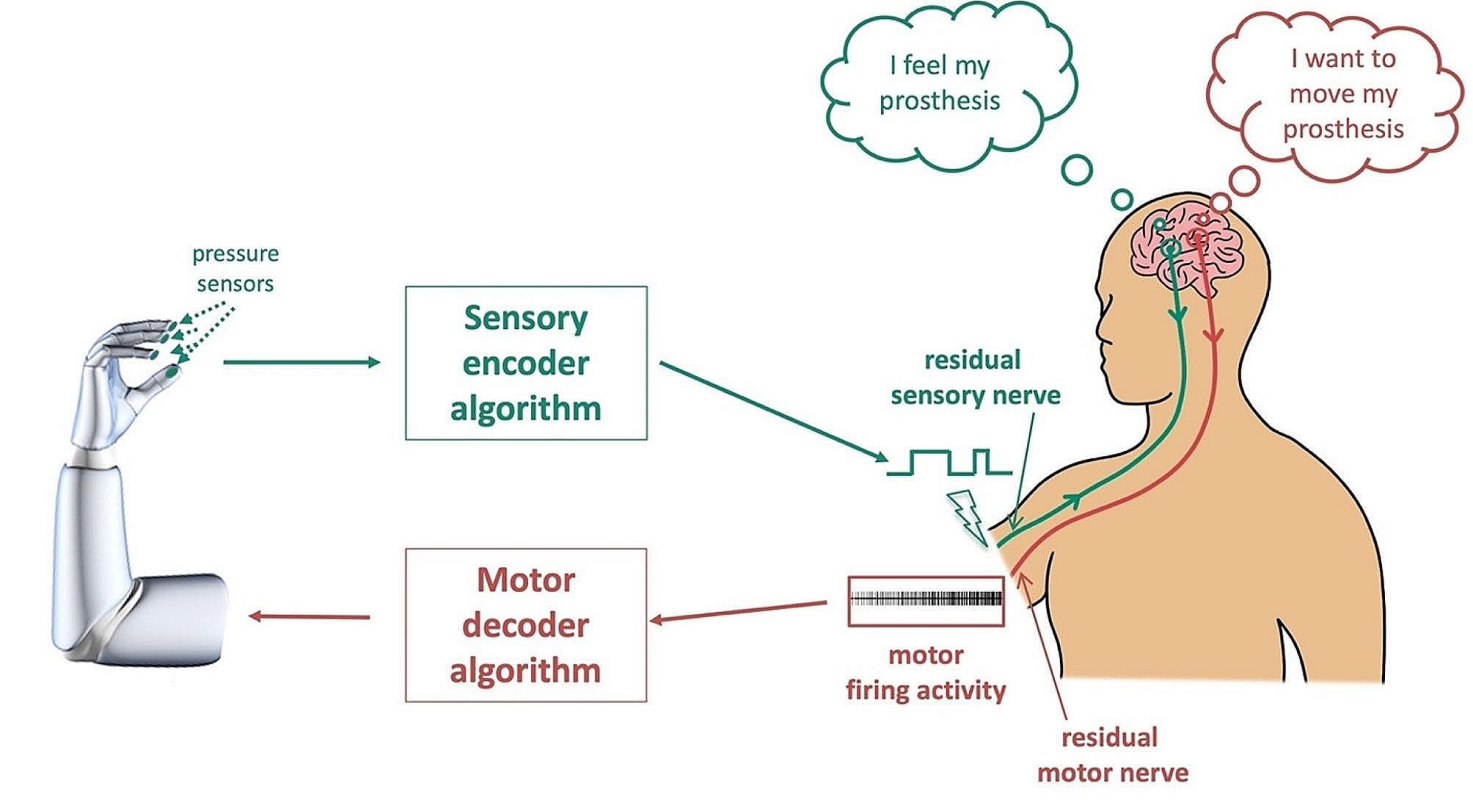
Bidirectional sensorimotor prosthetic control. Recorded motor activity from residual motor nerves in the amputee’s stump, which is proportional to the motor intent, drive – via a motor decoder – the prosthesis movement (red pathway). Simultaneously and independently, electrical signals from the prosthesis pressure sensors stimulate – via a sensory encoder – the residual sensory nerves in the stump to evoke the natural sensation of touch in the amputee’s sensory cortex (teal pathway). Figure adapted from [25]

SM neuroprostheses are advanced prosthetics that aim to restore sensory and motor functions by directly interfacing with the nervous system. These prostheses typically involve the use of implanted electrodes to separately connect with residual sensory and motor nerves (to provide sensory feedback and record the motor intent); thereby, enabling bidirectional communication between the brain and prosthesis to restore lost sensory and motor function due to injury, disease, or amputation (Fig. 3). To restore lost motor function, SM neuroprostheses measure the electrical activity of residual motor nerves in the amputee’s stump and convert that, via motor decoder algorithms, into a command signal that drives electric prosthesis motors (Fig. 3, red pathway). To restore lost sensory function, SM neuroprostheses, simultaneously and independently, send electrical signals from the prosthetic hand pressure sensors and convert them, via sensory encoder algorithms, into electrical stimulation waveforms of varying amplitude and frequencies. This electrical stimulation of residual sensory nerves in the amputee’s stump evokes a sensation of touch in the sensory cortex (Fig. 3, teal pathway). This sensory input converges back at the motoneuron to provide a sensory feedback signal. As such, SM neuroprostheses enable bidirectional communication of sensory information (to the nervous system) and motor commands (from neural signals) between the nervous system and the prosthesis. To evoke natural and intuitive sensations, sensory encoders should therefore reproduce normal patterns of neuronal activation [20]. This has been shown to be possible as sensation quality was shaped by gradation of the neural activation stimulation frequency (only up to 50 Hz) [21]. Thus, it is possible with the development of encoding strategies to convey touch feedback through prosthetics. In conditions when the connection between the brain and spinal cord is interrupted (e.g., after spinal cord injury or in neurodegenerative diseases), restoration of sensory and motor functions could be achieved via direct stimulation of and recording from the sensory and motor regions in the brain (through a brain-machine interface system), respectively, as opposed to stimulating/recording of peripheral sensory and motor nerves.

The primary advantages of SM neuroprostheses are the intuitive control of the prosthesis and the restoration of tactile sensation. Importantly, they enhance both functionality and aesthetics. Additionally, electrical stimulation for sensory restoration has been shown to reduce phantom pain [22] and helped amputees in prosthetic embodiment [23]. However, SM neuroprostheses require frequent daily calibration of the sensory encoder and motor decoder algorithms (to maintain responsive performance), have complicated software and hardware control mechanisms, could be expensive to repair [24].

### Neural plasticity after injury and SM interactions

Following amputation, cortical and spinal neuroplastic changes take place, which influence the recovery and utilization of SM neuroprostheses. Cortical reorganization is thought to occur across two distinct time frames: an initial acute phase, during which reorganization arises from the revealing of latent neural circuits, and a subsequent chronic phase marked by delayed reorganization, possibly attributed to axonal sprouting [26, 27]. The neuroplastic changes are not limited to cortical areas of the affected limb, but also extend to other brain areas and those of the intact limb. For instance, loss of afferent sensory information shifts the cortical representation of the affected limb as well as the homotopic cortical area in the opposite hemisphere [28, 29]. Also, Williams, Pirouz [29] noted an expansion of sensorimotor areas to more posterior parieto-occipital regions when performing a reaching task with the amputated limb, while use of their intact arm demonstrated higher ipsilateral sensorimotor activity. Generally, the changes in sensory representation do not affect the ability of amputees to perform tasks with their affected limb [28, 30]. In fact, such cortical changes have been associated with better performance on the intact limb’s motor tasks and are thought to be a functional adaptation [31, 32].

In the spinal cord, in animal models of nerve injury, spinal motoneurons have been shown to experience many cellular [33, 34], ionic [35], and structural changes after axotomy. Interestingly, on the latter, while axotomized spinal motoneurons experienced reductions in their dendritic trees [34], neck motoneurons exhibited expansion of their dendritic trees [36]. Axotomized spinal motoneurons also undergo significant alterations in their synaptic connections accompanied by a neuroinflammatory response, a reaction that is conserved across different types of motoneurons, injuries, and species [for review, see 37]. Importantly, axotomy is followed by a rapid decline in the neural output of axotomized spinal motoneurons [38, 39], which is not reversed or prevented by chronic electrical stimulation [38]. However, in humans, when amputees were exposed to artificial sensory feedback, task accuracy significantly improved, regardless of time since amputation [40]. Thus, while spinal and cortical reorganization results from loss of sensory inputs, the viability of efferent and afferent nerve pathways to the periphery are retained [41, 42].

After amputation, neuropathic pain also emerges causing phantom limb pain and residual limb pain after limb loss. Nearly 68% of amputees suffer from phantom limb pain, 42% suffer from residual limb pain, and 26% suffer from both [28]. The mechanisms underlying such postamputation pains are unclear [43]; however, residual limb pain is largely thought to result from neuroma formation (also known as neuroma pain) [43, 44]. Cellular changes causing hyperexcitability of dorsal root ganglion (DRG) sensory neurons are also thought to contribute to the emergence of pain after injury [45, 46]. Importantly, sensory stimulation has been shown to reduce phantom limb pain [47, 48], as well as improve the reintegration, embodiment, and controllability of the prosthesis [49].

### Challenges of prosthetics SM control under injury conditions

**Fig. 4:**
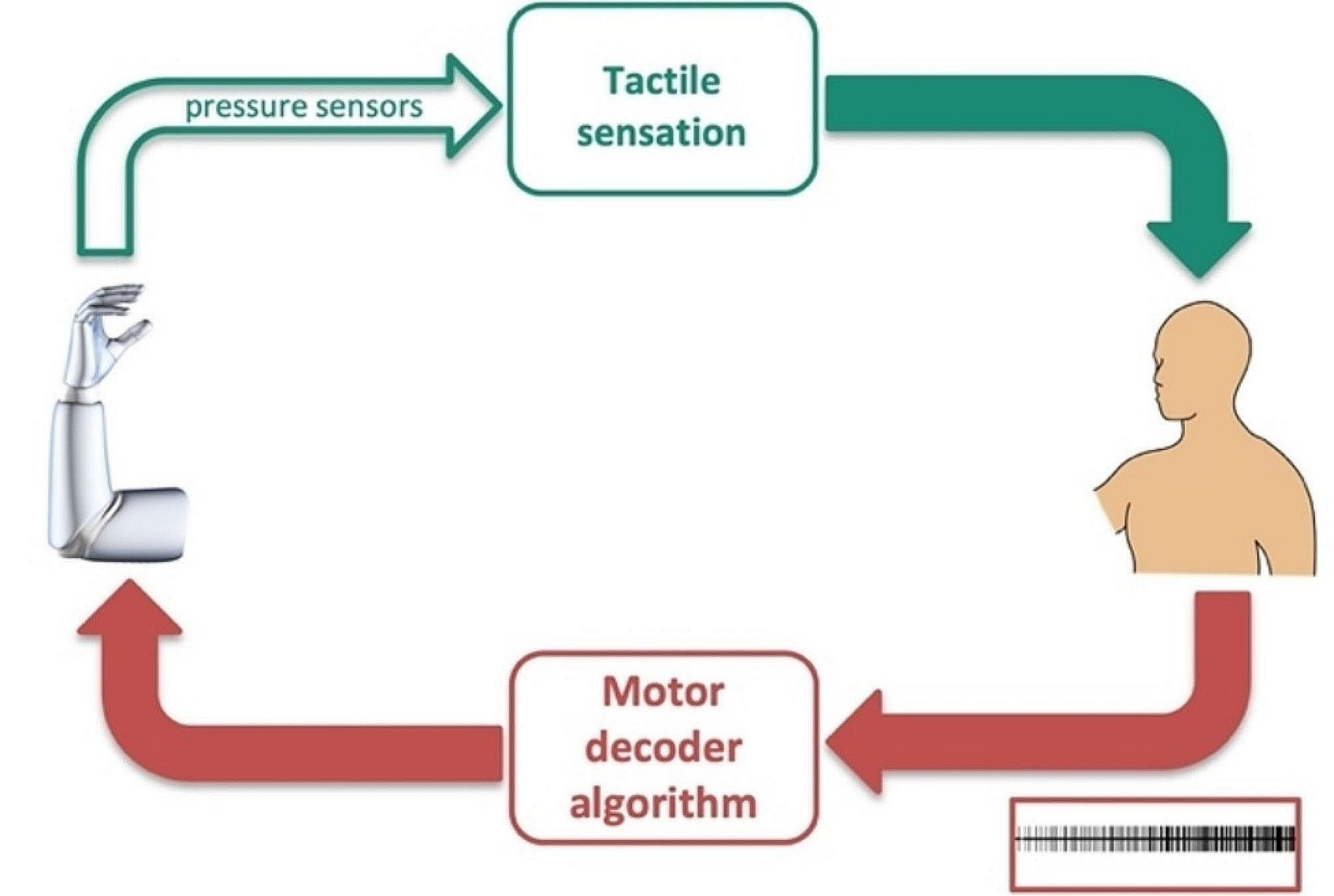
Optimizing the latency between the motor decoder and sensory encoder algorithms is crucial to restore true negative feedback SM prosthetic control

While current state-of-the-art prostheses are mechanically capable of sophisticated movements, they lack nuanced control signals and decoder/encoder algorithms to operate them at full capability. For instance, robotic arms and hands enable intricate manipulation beyond what can be achieved from volitional control [50]. Additionally, several key differences exist between movement control under healthy versus injury conditions that result in suboptimal SM control:

**1**) Because of the absence of amputated muscle as well as the preset, concurrent operation of the sensory encoder and motor decoder algorithms, the prosthetic movement control does not run in a true SM-coordinated scheme anymore – because the sensory input does not determine the motor consequences (Fig. 4). Because conduction velocities are different between sensory and motor axons (due to cellular and anatomical differences), the rates of sensory and motor information updates via the SM spinal pathways are very different. Accordingly, the latency of sensory encoder and motor decoder algorithms would be expected to also differ to restore SM coordination for better prosthetic performance.

**2**) Sensory stimulation under injury conditions is evoked via electrical stimulation of the sensory nerves, as opposed to muscle spindle firing when the intact muscle contracts. This difference might not result in evoking sensations of the full natural bandwidth. Also, figuring out electrical stimulation parameters (amplitudes, pulse widths, frequencies, and waveform shapes) that generate natural, as opposed to artificial, sensations is critical [21]. Searching the parameter space of these variables – both independently and in combinations – is also a challenge in itself. As a unique solution of optimal stimulation parameters is probably not present, employing artificial intelligence (AI) and machine learning methods could facilitate the identification of effective stimulation parameters that evoke sensations similar to those elicited by normal touch [51]. By generating synthetic data, these methods could augment limited sensory electrical stimulation evoked firing data obtained during calibration to enrich the searched datasets to identify optimal stimulation parameters. Generating subject-invariant data by these methods could also help expand the calibration datasets when the inter-subject variability is high (see Eldawlatly [52] for a recent review on the use of generative AI in brain-machine interface applications). Validating the neural responses of the AI/machine learning-driven stimulation algorithms would probably need to occur in animal experiments (rodents and non-human primates) before establishing the efficacy of those algorithms in evoking natural sensations in amputees.

**3**) Stimulation of severed sensory nerves and recordings from severed motor nerves by the prosthesis occur simultaneously, causing the motoneuron to be bombarded with repeated, concurrent sensory and motor synaptic inputs. This is different from the normal case (i.e., the healthy condition) when the motoneuron receives asynchronous sensory and motor synaptic inputs (due to the difference in conduction velocities between peripheral sensory and descending motor pathways). Understanding what motor output results from synchronized SM electrical stimulation is currently under investigation [53] but more research is needed to identify the optimal timing of stimulating the severed sensory and motor nerves. This information would be critical for generating seamless prosthetic movements.

**4**) Finally, current prostheses require several daily calibrations to adjust the motor decoder signal recording frequency and sensory encoder waveform amplitude and frequency settings to match the amputee’s changing neuromodulatory levels. Developing smart algorithms that adapt to the amputee’s changing neuromodulatory state is currently under investigation [54, 55] but more research is needed. Again, this is an area where AI and machine learning techniques could enhance the adaptability of the sensory encoder and motor decoder algorithms to the continuously changing amputee’s neuromodulatory state; thereby, minimizing the number of daily calibration sessions.

In sum, to engineer coordinated SM functionalities to operate a prosthesis, the aforementioned challenges would need to be addressed to accomplish true and optimized SM coordination.

### Future advances of SM prosthetics

Future SM neuroprostheses aim to restore all sensations as well as full motor functionality lost after amputation. While current effort has focused on the restoration of tactile sensation, other sensory modalities are important to also restore. For example, proprioception (through which the position and movement of own body parts are sensed), cutaneous, and thermal sensations are additional important sensations to include in prosthetics. Recently, natural thermal sensation was successfully restored in upper-limb amputees [56]. Also, prosthetics that involve multisensory integration have been recently used and have shown improved functional performance and better outcomes [47, 48, 57]. For instance, neuroprosthetics with multisensory stimulation that combined visual and tactile sensations allowed faster sensory processing [48], higher embodiment, and reductions in phantom limb pain [47, 48]. Also, prosthetics with position feedback (delivered via intraneural stimulation) combined with somatotopic tactile feedback allowed upper-limb amputees to regain high and close-to-natural remapped proprioceptive acuity with results comparable to healthy participants [57]. Combining the prosthesis position in space, speed of movement, pressure on the prosthetic, and the temperature of handled objects could help the prosthetic master control system mitigate potential risks to the amputee by averting hazardous movements. Nonetheless, integrating each sensory modality demands the development of its own sensory encoder.

**Fig. 5:**
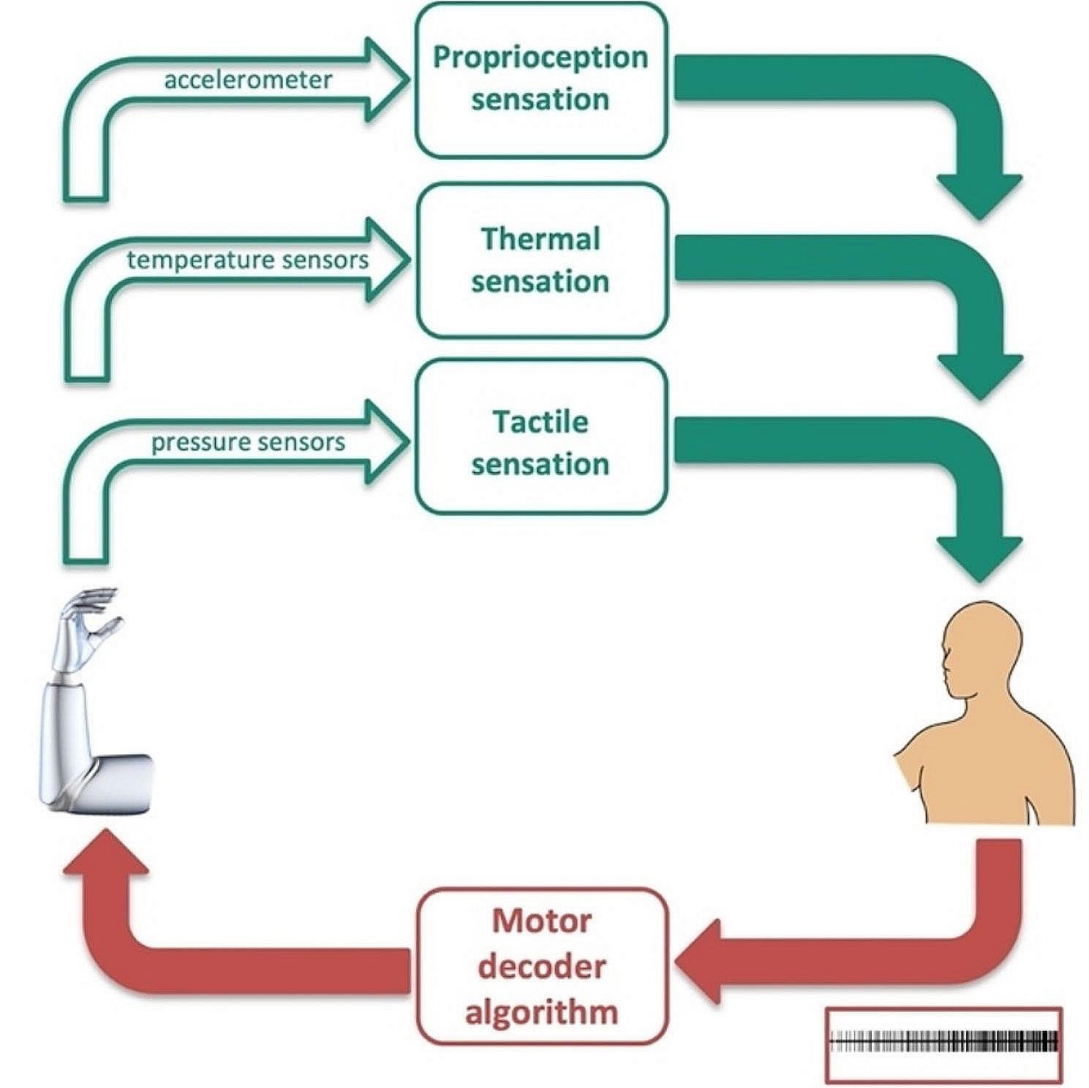
Restoration of multiple sensory modalities requires optimizing the latency between the motor decoder and each sensory encoder to achieve optimal prosthetic performance

With multisensory prosthetics, achieving effective coordination among various encoders and stimulation paradigms is imperative to ensure the nervous system’s independent and successful perception of each sensory mode (Fig. 5). Because the sensory pathways that mediate each sensation differ in their axonal diameters and conduction velocities, the latency of each sensory encoder and its information update rate would be expected to differ. Such intricate hardware and software complexity in prosthetic design and control presents formidable challenges that warrant extensive research endeavors for resolution and innovation.

## Conclusions

Although upper limb prostheses have come a long way, they are still unable to provide true SM coordination of the prosthetic movements as mimicking the SM neurophysiology and its integrated control is a hard task. The ability to drive the prosthesis in proportional to the motor intent, provide multiple modalities of sensory feedback, and optimize the latencies among the motor decoder and sensory encoder algorithms remain key milestones for accomplishing smart, advanced, and reliable prosthetic control. Employing AI and machine learning tools in control algorithms could greatly help the field reach those milestones; thereby, improving the quality of life of amputees.

## Data Availability

Not applicable.

## Abbreviations

AI: Artificial intelligence
SM: Sensorimotor
DRG: Dorsal root ganglion

## References

[1] Rydland J, et al. Neurorehabilitation in adults with traumatic Upper Extremity Amputation: a scoping review. Neurorehabil Neural Repair. 2022;36(3):208–16. 34967259 10.1177/15459683211070277

[2] Ziegler-Graham K, et al. Estimating the prevalence of limb loss in the United States: 2005 to 2050. Arch Phys Med Rehabil. 2008;89(3):422–9. 18295618 10.1016/j.apmr.2007.11.005

[3] Biddiss EA, Chau TT. Multivariate prediction of upper limb prosthesis acceptance or rejection. Disabil Rehabil Assist Technol. 2008;3(4):181–92. 19238719 10.1080/17483100701869826

[4] Carey SL, Lura DJ, Highsmith MJ. Differences in myoelectric and body-powered upper-limb prostheses: systematic literature review. J Rehabil Res Dev. 2015;52(3):247–62. 26230500 10.1682/JRRD.2014.08.0192

[5] Burke RE, Levine DN, Zajac FE 3. Mammalian motor units: physiological-histochemical correlation in three types in cat gastrocnemius. Science. 1971;174(4010):709–12. 10.1126/science.174.4010.709 4107849

[6] Lee RH, Heckman CJ. Adjustable amplification of synaptic input in the dendrites of spinal motoneurons. Vivo J Neurosci. 2000;20(17):6734–40. 10964980 10.1523/JNEUROSCI.20-17-06734.2000 PMC6772971

[7] Lee RH, Heckman CJ. Enhancement of Bistability in spinal motoneurons in vivo by the noradrenergic alpha 1 agonist methoxamine. J Neurophysiol. 1999;81(5):2164–74. 10322057 10.1152/jn.1999.81.5.2164

[8] Hounsgaard J, et al. Bistability of alpha-motoneurones in the decerebrate cat and in the acute spinal cat after intravenous 5-hydroxytryptophan. J Physiol. 1988;405:345–67. 3267153 10.1113/jphysiol.1988.sp017336 PMC1190979

[9] Binder MD, Heckman CJ, Powers RK. The physiological control of motoneuron activity. Section 12: Exercise: Regulation and Integration of Multiple Systems. New York: Oxford Univ; 1996. pp. 3–53.

[10] Lee RH, Heckman CJ. Influence of voltage-sensitive dendritic conductances on bistable firing and effective synaptic current in cat spinal motoneurons in vivo. J Neurophysiol. 1996;76(3):2107–10. 8890322 10.1152/jn.1996.76.3.2107

[11] Atallah H, Qureshi AZ, Msechu Z. Satisfaction of individuals with partial-hand amputations after they were fitted with cosmetic silicone prostheses. Prosthet Orthot Int. 2023;47(3):288–92. 36705663 10.1097/PXR.0000000000000196

[12] Capsi-Morales P, et al. Functional assessment of current upper limb prostheses: an integrated clinical and technological perspective. PLoS ONE. 2023;18(8):e0289978. 37585427 10.1371/journal.pone.0289978 PMC10431634

[13] Farina D, et al. The extraction of neural information from the surface EMG for the control of upper-limb prostheses: emerging avenues and challenges. IEEE Trans Neural Syst Rehabil Eng. 2014;22(4):797–809. 24760934 10.1109/TNSRE.2014.2305111

[14] Yadav D, Veer K. Recent trends and challenges of surface electromyography in prosthetic applications. Biomed Eng Lett. 2023;13(3):353–73. 37519867 10.1007/s13534-023-00281-z PMC10382439

[15] Mesin L. Optimal spatio-temporal filter for the reduction of crosstalk in surface electromyogram. J Neural Eng. 2018;15(1):016013. 28948938 10.1088/1741-2552/aa8f03

[16] Kuiken TA, et al. Targeted muscle reinnervation for real-time myoelectric control of multifunction artificial arms. JAMA. 2009;301(6):619–28. 19211469 10.1001/jama.2009.116 PMC3036162

[17] Aszmann OC, Dietl H, Frey M. *[Selective nerve transfers to improve the control of myoelectrical arm prostheses]* Handchir Mikrochir Plast Chir, 2008. 40(1): pp. 60 – 5. 10.1055/s-2007-989415 18322900

[18] Cheesborough JE, et al. Targeted muscle reinnervation and advanced prosthetic arms. Semin Plast Surg. 2015;29(1):62–72. 25685105 10.1055/s-0035-1544166 PMC4317279

[19] Wang B et al. Surface EMG Statistical and Performance Analysis of targeted-muscle-reinnervated (TMR) transhumeral prosthesis users in Home and Laboratory settings. Sens (Basel), 2022. 22(24). 10.3390/s22249849 PMC9786766 36560218

[20] Bensmaia SJ, Tyler DJ, Micera S. Restoration of sensory information via bionic hands. Nat Biomed Eng. 2023;7(4):443–55. 33230305 10.1038/s41551-020-00630-8 PMC10233657

[21] Graczyk EL, et al. Frequency shapes the quality of Tactile Percepts evoked through Electrical Stimulation of the nerves. J Neurosci. 2022;42(10):2052–64. 35074865 10.1523/JNEUROSCI.1494-21.2021 PMC8916769

[22] Soghoyan G, et al. Peripheral nerve stimulation enables somatosensory feedback while suppressing phantom limb pain in transradial amputees. Brain Stimul. 2023;16(3):756–8. 37100202 10.1016/j.brs.2023.04.017

[23] Wang W, et al. Neuromorphic sensorimotor loop embodied by monolithically integrated, low-voltage, soft e-skin. Science. 2023;380(6646):735–42. 37200416 10.1126/science.ade0086

[24] Dantas H, et al. Deep Learning Movement intent decoders trained with dataset aggregation for prosthetic limb control. IEEE Trans Biomed Eng. 2019;66(11):3192–203. 30835207 10.1109/TBME.2019.2901882

[25] Elbasiouny SM. *Cross-Disciplinary Medical Advances with Neuroengineering: Challenges Spur Development of Unique Rehabilitative and Therapeutic Interventions*, in *IEEE Pulse*. 2017, IEEE. pp. 4–7. 10.1109/MPUL.2017.2729739 28961088

[26] Li Y, et al. Cortical functional reorganization in response to intact forelimb stimulation from acute to chronic stage in rodent amputation model. Annu Int Conf IEEE Eng Med Biol Soc. 2018;2018:21–4. 30440331 10.1109/EMBC.2018.8512218

[27] Pearson PP, et al. Delayed reorganization of the shoulder representation in forepaw barrel subfield (FBS) in first somatosensory cortex (SI) following forelimb deafferentation in adult rats. Exp Brain Res. 2003;153(1):100–12. 12955377 10.1007/s00221-003-1625-z

[28] Valyear KF, et al. Interhemispheric transfer of post-amputation cortical plasticity within the human somatosensory cortex. NeuroImage. 2020;206:116291. 31639508 10.1016/j.neuroimage.2019.116291 PMC6980947

[29] Williams L, et al. Remodeling of cortical activity for motor control following upper limb loss. Clin Neurophysiol. 2016;127(9):3128–34. 27472549 10.1016/j.clinph.2016.07.004 PMC4980263

[30] Schabowsky CN, et al. Trans-radial upper extremity amputees are capable of adapting to a novel dynamic environment. Exp Brain Res. 2008;188(4):589–601. 18443766 10.1007/s00221-008-1394-9

[31] Philip BA, Frey SH. Compensatory changes accompanying chronic forced use of the nondominant hand by unilateral amputees. J Neurosci. 2014;34(10):3622–31. 24599461 10.1523/JNEUROSCI.3770-13.2014 PMC3942579

[32] Makin TR, et al. Deprivation-related and use-dependent plasticity go hand in hand. Elife. 2013;2:e01273. 24220510 10.7554/eLife.01273 PMC3823186

[33] Yamuy J, et al. Passive electrical properties of motoneurons in aged cats following axotomy. Brain Res. 1992;570(1–2):300–6. 1617420 10.1016/0006-8993(92)90594-y

[34] Mentis GZ, et al. Early alterations in the electrophysiological properties of rat spinal motoneurones following neonatal axotomy. J Physiol. 2007;582(Pt 3):1141–61. 17510183 10.1113/jphysiol.2007.133488 PMC2075252

[35] Kelly ME, et al. Axotomy affects calcium-sensitive potassium conductance in sympathetic neurones. Neurosci Lett. 1986;67(2):163–8. 2425300 10.1016/0304-3940(86)90391-5

[36] Rose PK, Odlozinski M. Expansion of the dendritic tree of motoneurons innervating neck muscles of the adult cat after permanent axotomy. J Comp Neurol. 1998;390(3):392–411. 9455900 10.1002/(sici)1096-9861(19980119)390:3<392::aid-cne7>3.0.co;2-x

[37] Alvarez FJ, et al. Synaptic plasticity on motoneurons after Axotomy: a necessary change in paradigm. Front Mol Neurosci. 2020;13:68. 32425754 10.3389/fnmol.2020.00068 PMC7203341

[38] Gordon T, et al. Axotomy-induced changes in rabbit hindlimb nerves and the effects of chronic electrical stimulation. J Neurosci. 1991;11(7):2157–69. 2066780 10.1523/JNEUROSCI.11-07-02157.1991 PMC6575471

[39] Gordon T, et al. Long-term effects of axotomy on neural activity during cat locomotion. J Physiol. 1980;303:243–63. 7431233 10.1113/jphysiol.1980.sp013283 PMC1282889

[40] Schiefer MA, et al. Artificial tactile and proprioceptive feedback improves performance and confidence on object identification tasks. PLoS ONE. 2018;13(12):e0207659. 30517154 10.1371/journal.pone.0207659 PMC6281191

[41] Kuiken TA, et al. Targeted reinnervation for enhanced prosthetic arm function in a woman with a proximal amputation: a case study. Lancet. 2007;369(9559):371–80. 17276777 10.1016/S0140-6736(07)60193-7

[42] Lipschutz RD, et al. Shoulder disarticulation externally powered prosthetic fitting following targeted muscle reinnervation for improved myoelectric control. J Prosthetics Orthot. 2006;18(2):28–34. 10.3109/03093640409167756 15658637

[43] G DIP, et al. Neurophysiological models of phantom limb pain: what can be learnt. Minerva Anestesiol. 2021;87(4):481–7. 33432796 10.23736/S0375-9393.20.15067-3

[44] Treede RD, et al. Neuropathic pain: redefinition and a grading system for clinical and research purposes. Neurology. 2008;70(18):1630–5. 18003941 10.1212/01.wnl.0000282763.29778.59

[45] Abdulla FA, Smith PA. Axotomy- and autotomy-induced changes in the excitability of rat dorsal root ganglion neurons. J Neurophysiol. 2001;85(2):630–43. 11160499 10.1152/jn.2001.85.2.630

[46] Abdulla FA, Smith PA. Changes in na(+) channel currents of rat dorsal root ganglion neurons following axotomy and axotomy-induced autotomy. J Neurophysiol. 2002;88(5):2518–29. 12424291 10.1152/jn.00913.2001

[47] Rognini G, et al. Multisensory bionic limb to achieve prosthesis embodiment and reduce distorted phantom limb perceptions. J Neurol Neurosurg Psychiatry. 2019;90(7):833–6. 30100550 10.1136/jnnp-2018-318570 PMC6791810

[48] Risso G, et al. Multisensory stimulation decreases phantom limb distortions and is optimally integrated. iScience. 2022;25(4):104129. 35391829 10.1016/j.isci.2022.104129 PMC8980810

[49] Bumbaširević M, et al. The current state of bionic limbs from the surgeon’s viewpoint. EFORT Open Rev. 2020;5(2):65–72. 32175092 10.1302/2058-5241.5.180038 PMC7047902

[50] Farina D, et al. Toward higher-performance bionic limbs for wider clinical use. Nat Biomed Eng. 2023;7(4):473–85. 34059810 10.1038/s41551-021-00732-x

[51] Rahiminejad E, et al. A Biomimetic Circuit for Electronic Skin with Application in Hand Prosthesis. IEEE Trans Neural Syst Rehabil Eng. 2021;29:2333–44. 34673491 10.1109/TNSRE.2021.3120446

[52] Eldawlatly S. On the role of generative artificial intelligence in the development of brain-computer interfaces. BMC Biomed Eng. 2024;6(1):4. 38698495 10.1186/s42490-024-00080-2 PMC11064240

[53] Mahrous AA, Mousa MH, Elbasiouny SM. The mechanistic basis for successful spinal cord stimulation to generate steady motor outputs. Front Cell Neurosci. 2019;13:359–74. 31456665 10.3389/fncel.2019.00359 PMC6698793

[54] Montgomery AE, Allen JM, Elbasiouny SM. Adaptive neural decoder for prosthetic hand control. Front Neurosci. 2021;15:590775. 33897340 10.3389/fnins.2021.590775 PMC8060566

[55] Gamal M, et al. In-silico development and assessment of a Kalman filter motor decoder for prosthetic hand control. Comput Biol Med. 2021;132:104353. 33831814 10.1016/j.compbiomed.2021.104353 PMC9887730

[56] Iberite F, et al. Restoration of natural thermal sensation in upper-limb amputees. Science. 2023;380(6646):731–5. 37200444 10.1126/science.adf6121

[57] D’Anna E et al. A closed-loop hand prosthesis with simultaneous intraneural tactile and position feedback. Sci Robot, 2019. 4(27). 10.1126/scirobotics.aau8892 33137741

